# A Case of Fracture of the Cranium with Loss of Brain

**Published:** 1840-08

**Authors:** A. H. Buchanan

**Affiliations:** Columbia, Tennessee


Art. III.—*A case of Fracture of the Cranium, with wound of the Dura Mater and loss of Brain.* By A. H. Buchanan, M.D., of Columbia, Tennessee.

On the 27th of August 1824, a boy aged 8 years, was found in a horse lot apparently lifeless, with a deep wound in the left temple, extending from the anterior part of the ear to the external corner of the eye. It was supposed, that he had been kicked on the head by one of the horses in the lot. I saw him about six hours after the accident, and upon examination found the temporal muscle greatly lacerated and bruised, and entirely cut through, down to the subjacent bone; and discovered in the wound several small portions of brain. The left eye was much injected with blood, and the eye-lids were swollen and livid, and the pupils widely dilated. The pulse was full and slow, and the patient slightly comatose. Upon dilating the wound with the scalpel, I found the anterior inferior angle of the parietal bone, and the adjoining portions of the temporal and frontal bones, to the size of half a dollar, fractured and depressed. The dura mater was pierced by a spicula of bone, and several portions of brain, about the size of a pea, were perceived lying on its surface. The cheek bone where it unites with the zygomatic process of the temporal bone, was also fractured and depressed. The portions of cranium which were depressed being entirely loose, were removed by a pair of forceps; which left exposed the wounded dura mater, the middle artery of which now bled freely, but soon ceased without any application. The cheek bone being properly adjusted, the lips of the wound were brought together by three stitches, and by adhesive strips; a compress was placed upon the wound and retained by a bandage drawn firmly about the head.

The boy complained but very little during the operation—being during the greater part of the time in a state of stupor. After he was put to bed he remained in a comatose condition for two days, with full, slow pulse, about 60 in the minute, and pupils dilated; but during this time, if roused up he recognised his acquaintances, and was sensible to all external impressions. He was bled freely, on the second day, and purged with Epsom salts; during the following night he complained at times, of some pain in the wound. Suspecting that the pressure from the compress and bandage might be too great, it was diminished a little, and in the morning on raising a corner of the adhesive strips at the lower end of the wound, a free discharge of pus took place. The pulse being full and slow he was again bled, and also purged with salts. The accumulation of pus in the wound occasionally produced some pain, but when he complained it was always evacuated, which relieved him. From this time, by keeping the bowels open with salts or oil, and restricting him to the lightest of diet, he gradually recovered, and in four weeks was walking about the house. No untoward symptom occurred afterwards.

The boy, I suppose, lost about half an ounce of brain, which as far as I could discover did not seem to affect his intellectual faculties, and his father is of opinion, that his mind is not injured. His mother, however, is quite sure, that his memory is not so good as before the accident. The case I consider worthy of reporting, because it gives additional evidence to the cases already on record, that a wound of the dura mater, with loss of brain, is not necessarily fatal, nor always followed by fungus cerebri. It is highly probable that the firm support maintained by the compress and bandage, in this case, during the cure, and even for several weeks after the external wound had healed, was the most efficient remedy in preventing the fungous growth so apt to occur under such circumstances.

*July*, 1840.

